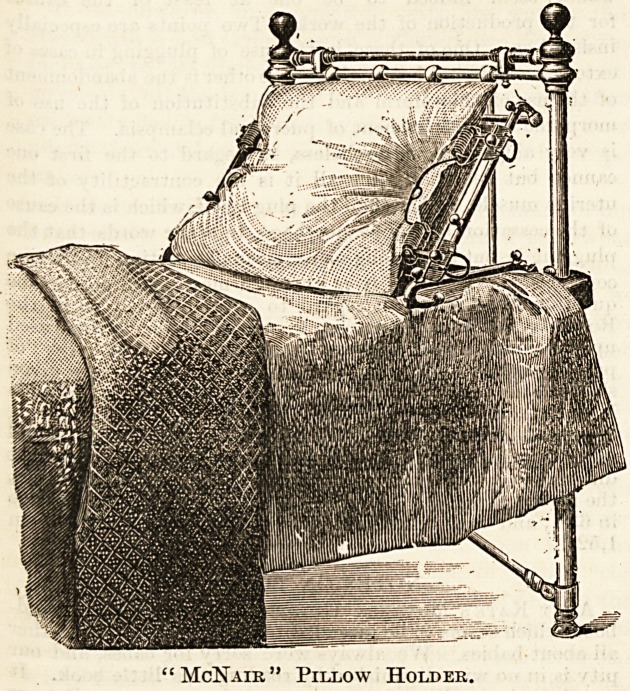# New Appliances and Things Medical

**Published:** 1899-08-12

**Authors:** 


### NEW APPLIANCES AND THINGS MEDICAL.

[We shall be glad to receive, at our Office, 28 & 29, Southampton Street, Strand, London, W.C., from the manufacturers, specimens of all new preparations and appliances which may be brought out from time to time.]

### "McNAIR" PILLOW HOLDER.

(R. Strang, Montpellier, Cheltenham.)

The above patent is a combined pillow-holder and bed rest. The pillow is held in position by two clips at each end, and it rests on a wire-wove mesh. The pillow-holder may be inclined at any angle, or set in the horizontal position. The

"McNair" Pillow Holder.

main advantages of this device are: The pillow is always held in position by means of the clips, and owing to the spring mesh on which it rests, it has greater elasticity and "give" than can be secured by any other means. When used as a bed-rest it can be inclined at any angle, and with the addition of one or two other pillows affords a most luxurious support for the back; by placing the top pillow above the level of the holder, and allowing it to rest against the back of the bed, comfortable support for the head is secured. The mechanism is throughout of the simplest description, and the illustration so self-explanatory that further details are unnecessary. The holder has already been much appreciated by those who have tried it, and bed-ridden invalids and persons affected with restlessness at night or insomnia will be well advised to give it a trial. The only improvement that we can suggest is that when in use in the horizontal position some protection to the head should be afforded against the oblique rails, which, bristling with knobs, would be anything but pleasant obstacles to strike one's head against by any sudden or unconscious movement.

### STOWER'S LIME JUICE CORDIAL.

(Alexander Riddle and Co., 36, Commercial Street, London, E.)

We have had a sample of the above cordial submitted to us for examination. It possesses the distinctive odour of the fresh fruit and the organic and inorganic salts which constitute the expressed juice. It may, therefore, be regarded chemically as an article of purity and the juice of the lime. The value of the juices of such fruits as the orange, the lemon, and the lime as anticorbutic elements is well known to the lay public; but in the medical mind the efficacy of the contained salts as eliminants of uric acid is held in no less esteem, and this is especially so in case of gout, rheumatism, and the uric acid diathesis. Practical experience shows that in such cases as these alkaline medicines or medicinal waters are to be prescribed which show on analysis a mineral content of the closest chemical affinity with that of the expressed juice of the above fruits, and it is easy to see that this is a far less elegant way of prescribing the medicaments than by ordering a free imbibition of lime juice cordial. In hot weather, or in localities where large quantities of fluid must be taken into the system to replace that which is lost by surface evaporation, there is no more health-giving beverage than pure water or soda water to which a modicum of lime juice has been added. And where lime juice is a desideratum, that which is prepared in the form of cordial and known as Stower's may be relied upon as first-rate, if not the best procurable.

## Figures and Tables

**Figure f1:**